# High ROR2 expression in tumor cells and stroma is correlated with poor prognosis in pancreatic ductal adenocarcinoma

**DOI:** 10.1038/srep12991

**Published:** 2015-08-11

**Authors:** Jianfei Huang, Xiangjun Fan, Xudong Wang, Yuhua Lu, Huijun Zhu, Wei Wang, Shu Zhang, Zhiwei Wang

**Affiliations:** 1 Department of Pathology, Nantong University Affiliated Hospital, Nantong, Jiangsu 226001, China; 2 Department of General Surgery, Nantong University Affiliated Hospital, Nantong, Jiangsu 226001, China; 3 Surgical Comprehensive Laboratory, Nantong University Affiliated Hospital, Nantong, Jiangsu 226001, China

## Abstract

RTK-like orphan receptor 2 (ROR2) is overexpressed in several cancers and has tumorigenic activity. However, the expression of ROR2 and its functional and prognostic significance have yet to be evaluated in pancreatic ductal adenocarcinoma (PDAC). Quantitative real-time polymerase chain reaction was used to characterize the expression of ROR2 mRNA in PDAC, corresponding peritumoral tissues, and PDAC cell lines. Immunohistochemical analysis with tissue microarrays was used to evaluate ROR2 expression in PDAC and to investigate the relationship of this expression to clinicopathological factors and prognosis. The expression of ROR2 mRNA and protein was significantly higher in PDAC than in normal pancreatic tissues. High cytoplasmic ROR2 expression in cancer cells was significantly associated with a primary tumor, distant metastasis, and TNM stage, and high stromal ROR2 expression was significantly associated with regional lymph node metastasis and TNM stage. The Kaplan–Meier method and Cox regression analyses showed that high ROR2 expression in tumor cytoplasm or stromal cells was significantly associated with malignant attributes and reduced survival in PDAC. We present strong evidence that ROR2 could be used as an indicator of poor prognosis and could represent a novel therapeutic target for PDAC.

Pancreatic ductal adenocarcinoma (PDAC) is one of the most lethal malignancies; it is the fourth leading cause of cancer-related mortality in the USA^1^ and the seventh leading cause of cancer-related death worldwide^2^. It is responsible for >227,000 deaths each year globally^3^,^4^. As a result of the absence of effective early detection methods and the lack of useful biomarkers for PDAC, >85% of tumors have already extended beyond the organ margins at the time of diagnosis and have invaded the perineural spaces within and beyond the pancreas, thus leading to the devastatingly poor outcomes of PDAC patients^5^,^6^. For now, surgical resection is the only curative treatment strategy. The prognosis for patients who undergo resection of PDAC with curative intent is generally poor, and relapse within 2 years occurs in >80% of patients^7^,^8^,^9^,^10^. For patients with advanced pancreatic cancer, systemic gemcitabine-based chemotherapy has been widely used as a standard therapy^11^. However, the long-term efficacy and prognosis still varies and is unsatisfactory among patients with PDAC^12^. Despite the massive development of therapeutic strategies, the prognosis for PDAC has barely changed for the past 25 years; the overall 5-year survival rate remains <4%^2^. Thus, there is an urgent need for the establishment of useful prognostic markers and novel potential therapeutic targets to facilitate the diagnosis and the rational treatment of PDAC.

Receptor tyrosine kinases (RTKs) are cell surface receptors that modulate normal cellular processes through ligand-controlled tyrosine kinase activity. RTKs have been reported to play a significant role in cancer development, and RTKs have been identified as therapeutic targets for certain types of cancer^13^. RTK-like orphan receptor (ROR)2 is an orphan receptor that is expressed in the developing embryo and is present in the embryonic limb buds, heart, primitive genitalia, developing somites, and mesenchymal cells^14^. ROR2 expression is necessary to regulate cell migration during palate development in mammals, and mutations in ROR2 can lead to diseases such as brachydactyly type B and Robinow syndrome^15^. Recently, several studies have demonstrated that ROR2 acts as a receptor that regulates canonical and noncanonical Wnt signaling and plays a pivotal role in Wnt5a-induced filopodia outgrowth and cell metastasis^16^,^17^,^18^. Moreover, ROR2 is overexpressed in several cancers and has pro-tumorigenic activity^19^,^20^,^21^. However, the expression of ROR2 and its clinical characteristics, especially its prognostic role, have yet to be investigated in PDAC.

Although some authors have reported that ROR2 expression might serve as a convenient prognostic biomarker and a potential therapeutic target for malignant tumors^21^,^22^,^23^,^24^, they may have ignored ROR2 protein expression in tumor stromal cells. It is well known that epithelial–stromal interactions play an important role in cancer development^25^,^26^. High ROR2 protein expression in fibroblasts occurred in the PDAC cases in our study.

In the present study, we detected the expression of ROR2 mRNA in fresh PDAC tissue and cell lines via one-step quantitative reverse transcription-polymerase chain reaction (qRT-PCR). Subsequently, we investigated ROR2 expression in PDAC and its relationship with clinical parameters by tissue microarray (TMA) and immunohistochemistry (IHC) and comparison with adjacent tissues. Finally, we further analyzed the clinicopathological attributes of ROR2, including its prognostic significance in PDAC patient survival.

## Results

### ROR2 mRNA expression in PDAC cell lines and tissues

**Figure 1:**
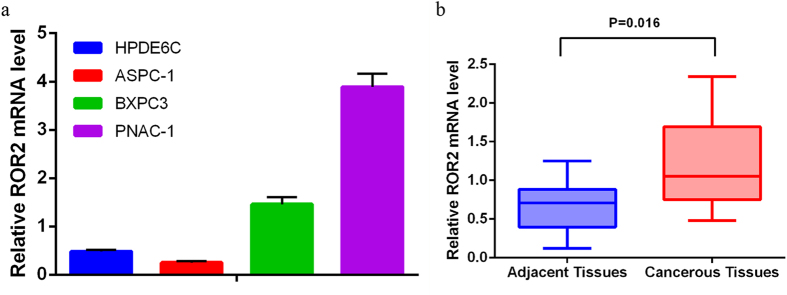
The mean qRT-PCR-based ROR2 mRNA expression normalized to 18S rRNA. Two PDAC cell lines (BXPC3, PNAC-1) have higher ROR2 expression than the benign pancreatic ductal cell line (HPDE6C7). The mean expression level of ROR2 mRNA in cancerous tissue was higher on average in the tumor tissues than in corresponding non-malignant tissues.

The relative expression of ROR2 mRNA in cell lines and tissues was quantified by qRT-PCR in replicates. When normalized to 18S rRNA, the mean expression levels of ROR2 mRNA in one benign pancreatic ductal cell line (HPDE6C7) and 3 PDAC cell lines (ASPC-1, BXPC3, PNAC-1) were 0.48 ± 0.04, 0.25 ± 0.03, 1.46 ± 0.15 and 3.89 ± 0.28, respectively (Fig. 1a). The mean expression levels of ROR2 mRNA in cancerous tissue (n = 10) and corresponding non-cancerous tissue (n = 10) were 1.226 ± 0.184 and 0.659 ± 0.107, respectively. The mean ROR2 expression in the tumor tissues was 1.886-fold higher than in non-malignant tissues (*P* *=* 0.016) (Fig. 1b).

### ROR2 protein expression in PDAC by IHC

**Figure 2:**
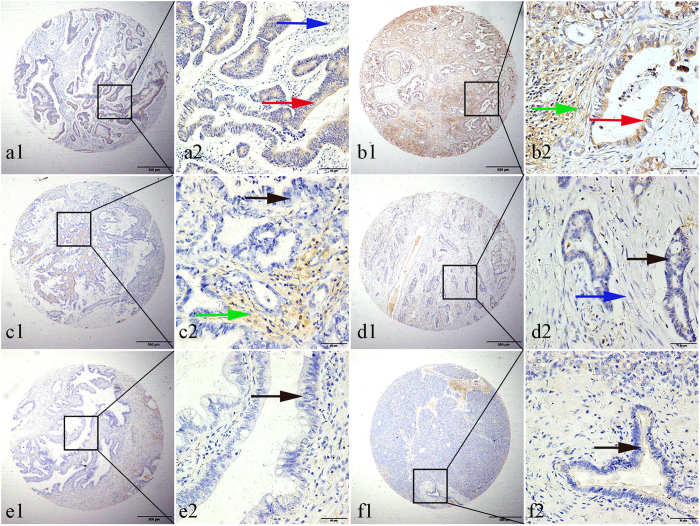
Representative pattern of ROR2 expression in pancreatic carcinoma (PDAC), benign pancreatic disease tissues, and adjacent noncancerous tissue as determined with TMA sections. (**a1**,**a2**) Positive tumor cytoplasmic (red arrow) and negative stromal (blue arrow) immunohistochemical staining of ROR2 in PDAC samples. (**b1**,**b2**) Strong tumor (red arrow) and stromal (green arrow) immunohistochemical staining of ROR2 in PDAC samples. (**c1**,**c2**) Negative IHC of ROR2 in tumor cells (black arrow) with strong positive stromal (green arrow) staining in PDAC tissue samples. (**d1**,**d2**) There was negative immunohistochemical staining of ROR2 in cancer cells (black arrow) and stromal cells (blue arrow). (**e1**,**e2**) Negative staining of ROR2 in epithelial cells (black arrow) in benign pancreatic disease tissues. (**f1**,**f2**) Negative immunohistochemical staining of ROR2 in benign pancreatic ductal cells (black arrow). Original magnification ×40 (bar = 500 μm) in (**a1**, **b1**, **c1**, **d1**, **e1** and **f1**); ×400 (bar = 50 μm) in (**a2**, **b2**, **c2**, **d2**, **e2** and **f2**).

We performed IHC analysis with TMAs to investigate ROR2 expression in PDAC (162 PDAC cases, 37 matched peritumoral tissue specimens, and 28 benign pancreatic disease specimens). Positive staining of ROR2 was primarily localized in the tumor cell cytoplasm and stromal cells of PDAC. All tumors could be classified into four subtypes according to ROR2 expression: (a) tumors with staining only in the tumor cells (cytoplasm) but not in stromal cells, (b) tumors with ROR2 expression in both compartments, (c) tumors with ROR2 confined to the tumor stroma, and (d) tumors without staining in tumor or stromal cells (Fig. 2). Moreover, for statistical analysis, the cutoff point for the ROR2 expression score was determined using X-tile software, with a statistically significant correlation between high ROR2 expression and patient outcome. First, we observed the staining percentages of positive cells, and then we recorded the staining intensity. The product of the intensity and percentage scores was used as the final ROR2 expression score. Subsequently, we performed univariate survival analysis with X-tile software based on the ROR2 expression score that we acquired previously. Then, several cutoff point values could be calculated, including 70%, 50% and 30%. We selected a cutoff that captures the ROR2 expression signal for the majority of TMAs by IHC analysis (i.e., ≥50%) and would likely yield more reproducible findings. When using a cutoff of 50% malignant and stromal cells in the TMA sample, we observed that our requirement for positivity did not change critically for the overall survival and was stable in X-tile. Thus, we employed this 50% cutoff as the criterion that best differentiated PDAC outcomes in the present study.

The degree of ROR2 expression was quantified using a two-level grading system, and expression scores were defined as follows: for ROR2 in tumor cells, low expression, 0–39, high expression, 40–300; for stromal cells, low expression, 0–59, high expression, 60–300.

High cytoplasmic expression of ROR2 was observed in PDAC tissue (74/162, 45.7%), matched peritumoral tissue (8/37, 21.6%), and benign pancreatic disease tissue (7/28, 25.0%) samples; the values in PDAC and peritumoral tissues differed significantly (χ^2^ = 10.019, *P* = 0.007). High stromal ROR2 expression was observed in 70 (43.2%) of the 162 PDAC samples, 14 (37.8%) of 37 matched peritumoral tissues, and 6 (21.4%) of 28 benign pancreatic disease tissues (χ^2^ = 4.79, *P* = 0.091). Typically observed IHC staining for ROR2 is shown in Fig. 2.

### Association between ROR2 expression and clinicopathological parameters of PDAC

The principal relationships of positive ROR2 expression with the clinicopathological variables of PDAC patients were further investigated. As shown in Table 1, high cytoplasmic ROR2 expression was significantly associated with primary tumor (*P* = 0.012), distant metastasis (*P* = 0.031), and TNM stage (*P* = 0.039), whereas high stromal ROR2 expression was significantly associated with regional lymph node invasion (*P* = 0.008) and TNM stage (*P* = 0.012). By contrast, no significant association was found between ROR2 expression and other clinical parameters, such as age and sex; tumor size, location, and differentiation; and vascular and extrapancreatic invasion (Table 1).

### Survival analysis

**Figure 3:**
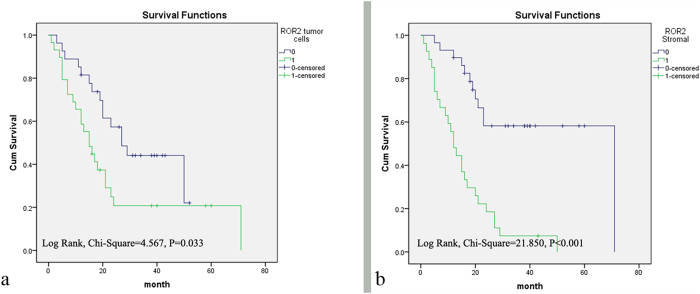
Survival analysis of PDAC patients by the Kaplan–Meier method and log-rank test. (**a**) Overall survival rate in patients with high cytoplasmic ROR2 expression (green line) was significantly lower than in patients with low or no cytoplasmic ROR2 expression (blue line). (**b**) Overall survival rate in patients with high stromal ROR2 expression (green line) was significantly lower than in patients with low or no stromal ROR2 expression (blue line).

Univariate analyses showed that increased cytoplasmic (*P* = 0.039) and stromal (*P* < 0.001) expression of ROR2 and regional lymph node invasion (*P* = 0.041) were associated with the 5-year survival rates of PDAC patients. Kaplan–Meier survival curves and multivariate analyses further demonstrated that cytoplasmic (*P* = 0.038) and stromal (*P* < 0.001) ROR2 expression levels were independent prognostic factors (Table 2, Fig. 3).

## Discussion

It is well known that Wnt signaling is essential for embryonic development and cellular processes, including differentiation, polarity, migration, invasion, adhesion, and survival, all of which are crucial components of tumorigenesis and metastasis. Thus, Wnt signaling plays a vital role in cancer^27^,^28^,^29^. It has been reported that genetic and epigenetic alterations of components of the canonical Wnt signaling pathway are a primary mechanism of cancer development^30^.

ROR2 is also believed to participate in the Wnt signaling pathway, which is composed of several extracellular effectors, membrane proteins, intracellular signal transducers, and nuclear gene regulators^30^,^31^. Recent evidence suggests that Wnt5a is the sole ligand for ROR2, and the function of ROR2/Wnt5a signaling varies under different conditions; ROR2 can drive noncanonical signaling pathways and inhibit canonical β-catenin-dependent Wnt signaling^32^. ROR2 appears to possess dual roles and can act to suppress or promote carcinogenesis in different cancer tissues. In the case of canonical (β-catenin-dependent) Wnt signaling, ROR2 expression is lost, supporting its role as a gatekeeper of the canonical pathway. Alternatively, in cancers driven by noncanonical (β-catenin-independent) Wnt signaling, ROR2 expression is increased and is thought to play a critical role in driving tumorigenesis^33^,^34^,^35^. For PDAC, several studies have reported that Wnt5a/JNK or Wnt5a/NFAT signaling plays an important role in PDAC development^36^,^37^,^38^. Wnt5a/JNK and Wnt5a/NFAT signaling is noncanonical Wnt signaling; therefore it is logical to assume that ROR2 acts as a promoter in PDAC carcinogenesis.

In the present study, our qRT-PCR data confirmed that ROR2 mRNA expression in PDAC cells was higher than in benign pancreatic ductal cells, and ROR2 mRNA in PDAC tissues with tumor stroma was also obviously higher than in matched tumor-adjacent tissue, which agrees with the ROR2 protein expression in PDAC based on IHC analysis. It should be noted that only 10 pairs of PDAC and matched tumor neighbor tissues were included for qRT-PCR. The sample size was relatively small. However, these samples were extracted from randomly selected patients, and our qRT-PCR results did reflect ROR2 mRNA expression variation among different samples. For example, in cancerous tissues, the maximum ROR2 mRNA expression (approximately 2.3) was more than 4-fold higher than the minimum ROR2 mRNA expression (0.5).

TMAs of PDAC specimens and IHC analysis confirmed the ROR2 protein expression was primarily localized in the tumor cytoplasm, in agreement with previous reports^21^,^22^,^23^,^24^. The high ROR2 protein expression was detected in 45.68% (74/162) of PDAC tissue samples, which was higher than in matched tumor-adjacent tissue and benign pancreatic disease tissues samples. The data confirmed our hypothesis and were consistent with previous studies that indicated high ROR2 expression in cancer tissues, such as in 52.4% of squamous cell carcinomas of the oral cavity^19^ and 51.5% of medulloblastomas^24^. The minor difference may be from the choice of cutoff point; these data all support the finding that ROR2 expression was higher in cancer tissues than in non-tumor tissues.

Moreover, high ROR2 expression in PDAC was correlated with certain clinicopathological parameters, including primary tumor, distant metastasis, and TNM stage. The data validated the pro-tumorigenic role of ROR2 and were in accord with studies that reported its oncogenic role in malignancies^21^,^22^,^23^,^24^. Furthermore, high ROR2 expression in tumor cells is an independent factor for patient outcome^23^,^24^. Our study showed a similar result, and we also confirmed that high ROR2 expression in stromal cells independently predicted unfavorable overall survival for PDAC patients, as elucidated with multivariate analysis.

Although stromal cell ROR2 expression was reported^39^, to the best of our knowledge, the associations between ROR2 expression in the stroma and clinical features in PDAC to determine its clinicopathologic significance have not been investigated. In this study, ROR2 expression in stromal cells was associated with regional lymph node metastasis and TNM stage. Because stromal cell types with genetic stability have been reported to play important roles in the tumor microenvironment, including drug resistance and tumor recurrence^40^, a growing number of researchers have specifically noted the expression of tumor markers in the tumor stroma and found it to be closely related to tumor progression and the patient’s prognosis^41^,^42^,^43^. Our study therefore suggests that ROR2 expression in the tumor microenvironment may be associated with the invasive activity of PDAC tumor cells. These findings suggest that ROR2 in both stromal cells and cancer cells might play an important role in the progression of PDAC. Further *in vitro* and *in vivo* studies using PDAC models are necessary to confirm our findings concerning the exact function and therapeutic target of ROR2.

In conclusion, we identified, perhaps for the first time, that ROR2 plays an essential role as a prognostic marker of survival in patients with PDAC. Our results are helpful in understanding the roles of ROR2 in the progression and development of PDAC. Furthermore, ROR2 could be considered a potential therapeutic target of PDAC.

## Methods

### PDAC cell lines and patient specimens

PDAC cell lines (BXPC3, ASPC-1, PNAC-1) and one benign pancreatic ductal cell line, HPDE6C7, were obtained from the cell bank of the Chinese Academy of Science in Shanghai, China. They were cultured in DMEM (Gibco, Invitrogen, Carlsbad, CA, USA) containing 10% fetal calf serum (FCS) at 37 °C and 5% CO_2_.

Fresh-frozen tumor (10 PDAC) and matched peritumoral tissue samples (n = 10) were collected from PDAC patients from the Department of Pathology, the Affiliated Hospital of Nantong University. Simultaneously, 162 formalin-fixed, paraffin-embedded PDAC tumor samples, 37 matched peritumoral tissue specimens, and 28 benign pancreatic disease specimens were also collected from the Department of Pathology, the Affiliated Hospital of Nantong University, from 2003 to 2013. All the cases were re-evaluated for grade and histological type by two independent pathologists (Huang J and Zhang S). All pertinent clinical data were collected simultaneously, including the following: sex (male, n = 94, female, n = 68) and age (≤60 years, n = 64, >60 years, n = 98); tumor size, location and differentiation; vascular, perineural and extrapancreatic invasion; lymph node metastasis; and TNM stage. None of the patients received radiotherapy, chemotherapy, or immunotherapy prior to surgery. The overall survival duration was the interval from the date of first biopsy to the date of death from disease. Patients who were alive at the last follow-up date were censored from the analysis. The average survival duration in the PDAC group was 21.9 ± 16.8 months. Written informed consent was obtained from the patients for publication of this study and any accompanying images. The study protocol was approved by the Ethics Committee of the Human Research Ethics Committee of the Affiliated Hospital of Nantong University, and all experiments were performed in accordance with approved guidelines of the Affiliated Hospital of Nantong University.

### Quantitative real-time polymerase chain reaction (qRT-PCR)

Total RNA was isolated from PDAC cell lines and a subset of fresh-frozen tissues (10 fresh-frozen PDAC tumor and matched peritumoral tissue samples) using the RNeasy Plus Mini Kit (74134, Qiagen, Germany) and converted to cDNA using the High-Capacity RNA-to-cDNA Kit (4387406, Life Technologies, USA). RNA quantity and quality were analyzed using a NanoDrop 1000 spectrophotometer. Real-time PCR was performed using a human ROR2 assay kit (4331182, Life, USA) and TaqMan Universal Master Mix II (4440038, Life, USA) on an ABI7500 system following the manufacturer’s instructions. The 18S rRNA (4453320, Life, USA) served as an endogenous control.

### TMA construction and IHC analysis

A total of 162 PDAC tissues, 37 matched peritumoral tissue specimens, and 28 benign pancreatic disease tissues were prepared and used in this study. We used the Tissue Microarray System (Quick-Ray, UT06, UNITMA, Korea) in the Department of Clinical Pathology, Nantong University Hospital, Jiangsu, China. Core tissue biopsies (2 mm in diameter) were taken from individual paraffin-embedded sections and arranged in the new recipient paraffin blocks. TMA specimens were cut into 4-μm sections and placed on super frost-charged glass microscope slides. IHC analysis was performed as previously described^44^. TMA slides were separately stained using polyclonal rabbit anti-ROR2 antibody (LifeSpan BioSciences Inc., Seattle, WA, USA). The secondary antibody used was horseradish-peroxidase-conjugated anti-rabbit antibody (Dako Cytomation, Carpinteria, CA, USA). For negative controls, phosphate-buffered saline was used instead of the primary antibody. Blind ROR2 immunostaining evaluation and independent observation were simultaneously performed. The expression of ROR2 was converted into continuous intensity values using the semiquantitative H-score method, based on both the staining intensity and the percentage of cells at that intensity. Briefly, the staining intensity was scored as 0 (−, no staining), 1 (+, weak staining), 2 (++, moderate staining), or 3 (+++, intense staining) using traditional semiquantitative pathology scoring. For each tissue sample, the percentage of cells stained at a certain intensity was determined and multiplied by the intensity score to generate an intensity percentage score. The final staining score of each tissue sample was the sum of the four intensity percentage scores, and these scores ranged from 0 (no staining) to 300 (100% of cells with +++ staining intensity). The cutoff point for the ROR2 expression score that was statistically significant in terms of survival was set using X-tile software (the Rimm lab at Yale University; http://www.tissuearray.org/rimmlab) as described previously^45^.

### Statistical analysis

The associations between ROR2 expression and important clinicopathological variables of PDAC patients were evaluated with χ^2^ tests. Factors shown to be of prognostic significance with the univariate Cox regression model were subsequently investigated with the multivariate Cox regression model. Survival curves were generated using the Kaplan–Meier method and log-rank test. For all tests, the significance level for statistical analysis was set at *P* < 0.05. All data were analyzed using STATA version 12.0 (Stata Corporation, College Station, TX, USA).

## Additional Information

**How to cite this article**: Huang, J. *et al.* High ROR2 expression in tumor cells and stroma is correlated with poor prognosis in pancreatic ductal adenocarcinoma. *Sci. Rep.* **5**, 12991; doi: 10.1038/srep12991 (2015).

## Figures and Tables

**Table 1:** Association of ROR2 expression with clinical attributes of pancreatic ductal adenocarcinoma.

Groups	No.	Cytoplasmic staining of ROR2 in tumor cells				Stromal staining of ROR2			
		Low or no expression (%)	High expression (%)	Pearson χ2	*P*value	Low or no expression	High expression (%)	Pearson χ2	*P*value
Total	162	88(54.32)	74(45.68)			92(56.79)	70(43.21)		
Age				0.159	0.690			0.013	0.911
≤60 years	64	36(56.25)	28(43.75)			36(56.25)	28(43.75)		
>60 years	98	52(53.06)	46(46.94)			56(57.14)	42(42.86)		
Sex				0.090	0.764			0.270	0.603
Male	94	52(55.32)	42(44.68)			55(58.51)	39(41.49)		
Female	68	36(52.94)	32(47.06)			37(54.41)	31(45.59)		
Tumor location				0.097	0.755			3.355	0.067
Head	85	46(54.12)	39(45.88)			43(50.59)	42(49.41)		
Body and/or tail	51	29(56.86)	22(43.14)			34(66.67)	17(33.33)		
Unknown	26	13	13			15	11		
Differentiation				0.044	0.833			1.840	0.175
Well and intermediate	126	69(54.76)	57(45.24)			68(53.97)	58(46.03)		
Poor	36	19(52.78)	17(47.22)			24(66.67)	12(33.33)		
Perineural invasion				0.748	0.387			2.772	0.096
no	14	9(64.29)	5(35.71)			10(71.43)	4(28.57)		
yes	64	33(51.56)	31(48.44)			30(46.88)	34(53.13)		
Unknown	84	46	38			52	32		
Vascular invasion				0.503	0.478			0.503	0.478
no	51	29(56.86)	22(43.14)			29(56.86)	22(43.14)		
yes	19	9(47.37)	10(52.63)			9(47.37)	10(52.63)		
Unknown	92	47	45			54	38		
T - Primary tumor				8.814	0.012*			4.634	0.099
T1–T2	86	55(63.95)	31(36.05)			55(63.95)	31(36.05)		
T3	54	26(48.15)	28(51.85)			28(51.85)	26(48.15)		
T4	20	6(30.00)	14(70.00)			8(40.00)	12(60.00)		
Unknown	2	1	1			1	1		
N – Regional lymph nodes				3.549	0.060			6.991	0.008*
N0	116	68(58.62)	48(41.38)			73(62.93)	43(37.07)		
N1	43	18(41.86)	25(58.14)			17(39.53)	26(60.47)		
Unknown	3	2	1			2	1		
M – Distant metastasis				4.671	0.031*			0.563	0.453
M0	152	85(55.92)	67(44.08)			87(57.24)	65(42.76)		
M1	7	1(14.29)	6(85.71)			3(42.86)	4(57.14)		
Unknown	3	2	1			2	1		
TNM stage				8.345	0.039*			10.882	0.012*
Stage 1a and stage 1b	66	42(63.64)	24(36.36)			47(71.21)	19(28.79)		
Stage 2a	36	21(58.33)	15(41.67)			19(52.78)	17(47.22)		
Stage 2b	37	17(45.95)	20(54.05)			16(43.24)	21(56.76)		
Stage 3 and stage 4	20	6(30.00)	14(70.00)			8(40.00)	12(60.00)		
Unknown	3	2	1			2	1		

*P < 0.05.

**Table 2:** Univariate and multivariate analysis of prognostic factors for 5-year survival in pancreatic cancer.

Variable	Univariate analysis			Multivariate analysis		
	HR	*P* value	95% CI	HR	*P* value	95% CI
Cytoplasmic expression of ROR2 high versus low	2.016	0.039*	1.037–3.919	2.103	0.038*	1.041–4.249
Stromal staining of ROR2 high versus low	4.641	<0.001*	2.274–9.475	4.702	<0.001*	2.172–10.181
Sex female versus male	1.137	0.710	0.578–2.233			
Age (years) ≤60 versus >60	0.940	0.854	0.486–1.819			
Tumor location head versus body and/or tail	0.667	0.292	0.314–1.418			
Differentiation well versus poor	1.720	0.180	0.778–3.802			
Perineural invasion yes versus no	1.490	0.589	0.350–6.334			
Vascular invasion yes versus no	1.225	0.658	0.499–3.008			
T T1-2 versusT3versus T4	1.009	0.964	0.669–1.524			
N N0 versus N1	2.008	0.041*	1.029–3.916	1.045	0.905	0.506–2.156
M M0 versus M1	0.738	0.767	0.099–5.495			
TNM stage I versus IIa versus IIb versus III–IV	1.029	0.840	0.777–1.364			

*P < 0.05.
